# Measles vaccination among children in border areas of Yunnan Province, Southwest China

**DOI:** 10.1371/journal.pone.0240733

**Published:** 2020-10-21

**Authors:** Jiangrong Li, Wenzhou Yu, Zhixian Zhao, Lei Zhang, Qiongyu Gong

**Affiliations:** 1 Expanded Program on Immunization Department, Yunnan Provincial Center for Disease Control and Prevention, Kunming, China; 2 National Immunization Program, Chinese Center for Disease Control and Prevention, Beijing, China; 3 Zhaotong Vocational College of Health, Zhaotong, China; The Chinese University of Hong Kong, HONG KONG

## Abstract

**Background:**

Border areas are at high risk of measles epidemics. This study aimed to evaluate the effectiveness of the implementation of the routine two-dose measles containing vaccine (MCV) program in border counties of Southwest China.

**Methods:**

Data used in the study were derived from a cross-sectional survey among 1,467 children aged 8 to 84 months from five border counties of Yunnan Province, Southwest China in 2016. The participants were recruited using a multistage sampling method. Primary guardians of the children were interviewed to collect information on vaccination history, socio-economic status, and knowledge about immunization. Both coverage and timely coverage for the first (MCV1) and the second (MCV2) dose of MCV were calculated. The Kaplan-Meier method was performed to estimate the cumulative coverage of MCV, and Log-rank tests were adopted to compare the differences across counties and birth cohorts. Univariate and multivariate logistic regression models were used to investigate the predictors of delayed MCV1 vaccination.

**Results:**

The coverage for MCV1 and MCV2 were 97.5% and 93.4%, respectively. However, only 63.8% and 84.0% of the children received MCV1 or MCV2 on time. Significant differences in the cumulative coverage were detected across counties and birth cohorts. Results of the multivariate logistic regression analysis indicated that children whose primary guardian knew the schedule of MCV were less likely to receive MCV1 late (OR = 0.63, P<0.01). For the guardians, doctors at vaccination units were the primary and also the most desired source of vaccination information.

**Conclusions:**

Although the coverage for MCV is high in border areas of Southwest China, the timeliness of MCV vaccination seems suboptimal. Tailored information from local health professionals may help to reduce untimely vaccination.

## Introduction

Measles is a highly contagious disease that causes enormous morbidity and mortality in many countries of the world [1]. At present, the most effective way to prevent and control measles epidemics is through Measles Containing Vaccine (MCV) vaccinations to ensure population immunity against measles virus [2]. Since 2005, China began to implement the two-dose policy of measles vaccination among children, and the annual incidence per 100,000 population dramatically decreased from 9.47 in 2005 to 0.46 in 2012 [3]. Although the national routine coverage of MCV has exceeded 95% over the past years, the timeliness of vaccination still remains low, even in some economically developed areas of China [4]. To further eliminate measles in China, it is of great significance to improve the implementations of the two-dose MCV vaccination program among children [5].

Border areas are usually at high risk of measles outbreaks [6, 7]. First of all, most border regions, especially in the Western China, are underdeveloped. Due to low levels of health awareness, insufficient access to vaccination services and other factors, the coverage and timeliness of MCV vaccination are reported to be lower in border areas [8, 9]. Moreover, policies and implementations of immunization programs vary substantially across countries. For instance, the national immunization coverage for MCV1 in Myanmar was lower than that in China in 2015 (86% versus 99%) [10, 11]. A study from China in 2017 also showed that measles antibody level among Myanmar migrants was lower than that among local residents [12]. Thus, it is very likely that the massive population movements across borders may enhance measles transmission across countries. For example, two measles outbreaks identified in border counties of Yunnan Province, Southwest China in 2009 and 2011 were mainly attributed to the population movements. One of them was caused by genotype D11 measles virus and the other was by genotype D9 [13].

As a border province in the Southwestern China, Yunnan shares 4,060 kilometers international border with Myanmar, Laos and Vietnam. Twenty five of the 129 counties in Yunnan Province are adjacent with one of the three countries. With the developments of international trade in recent years, interactions among border residents of these countries are substantially increasing. The purpose of this study was to assess the MCV coverage and its timeliness among children aged 8–84 months in border counties of Yunnan Province. By investigating the influential factors of vaccination behaviors, we also aimed to explore possible measures to improve MCV vaccination.

## Materials and methods

### Setting

**Fig 1:**
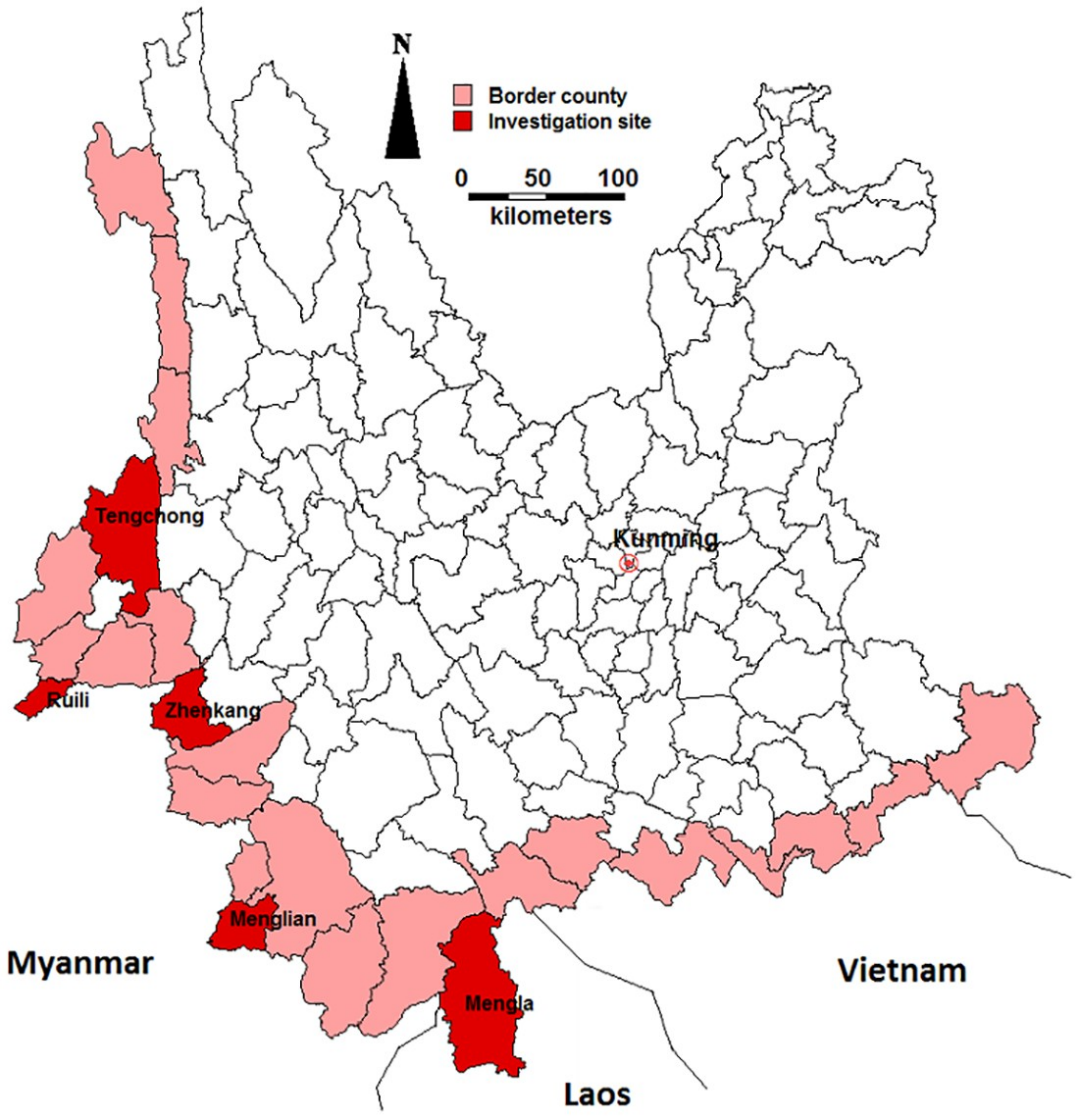
Geological location of the investigation sites. (Map generated with MapInfo 7.8 by authors).

Yunnan Province is located in Southwest China, with a population of 47 million in 2017. There are over 20 ethnic minorities living in Yunnan, with most of them residing in the border regions. Up to now, Yunnan Province has established 24 border ports with the number of inbound and outbound migrants reaching 45 million in 2018. At the provincial level, from 2013 to 2015, the coverage of MCV1 was 99.7%, 99.8% and 99.8%, while the annual incidence of measles in cases per 100 000 population was 3.04, 0.93 and 0.86, respectively. This study was conducted in 2016 in five of the 25 border counties in Yunnan Province, including Tengchong, Ruili, Zhenkang, Menglian and Mengla (Fig 1).

### Study design

The subjects in this study were children aged 8 to 84 months who had lived in the selected counties for at least three months. Children were excluded if they were not local residents or had been living in the selected counties for less than 3 months. A two-stage cluster sampling method recommended by the World Health Organization (WHO) was used to obtain a representative sample. First, five townships were randomly selected from each of the five counties. Second, six villages were randomly selected from each of the 25 selected townships. Altogether, 150 villages were selected as clusters. The sample size was estimated based on the following four parameters: an assumed MCV coverage of 90%, a desired precision of 5%, and a significance level of 0.05 and a design effect level of 2. The minimum sample size required for each county was 287. This number was increased to 300 to cover for a 5% non-response rate. Finally, 10 children per cluster for 150 clusters were determined as our sample size. A list of households with eligible children was obtained from local authorities in each selected village. Ten households were randomly selected from the list of each village. Only one child per household was selected to avoid clustering. When there were two or more eligible children in the same household, the youngest child would be selected following the WHO manual [14]. To ensure the age representativeness, at least one child in each age group was recruited. If there were not enough children in a selected village, the remaining children would be selected from the nearest village.

Primary guardians of the selected children (e.g. parents and grandparents) were invited to participate in the study. They were interviewed by trained health workers at local township health centers. A standard questionnaire was used to collect primary guardians’ sociodemographic characteristics, as well as their knowledge and attitude regarding immunization. The questionnaire consisted of three parts: (1) socio-demographic variables of primary guardians, such as gender, age, ethnicity, education levels, and annual household income per capita; (2) primary guardians’ knowledge and attitude regarding immunization, such as do they think MCV vaccination is important and safe for children, and do they know the schedule of MCV vaccination; (3) how do they get vaccination information. Vaccination status of each child was exclusively measured by parent-held vaccination certificates or cards. Self-reported receipt of vaccine but without written documentation was regarded as unknown vaccination status. In total, 1500 targeted children and their guardians completed the survey, among which 1467 were older than 8 months and 1340 were older than 24 months, respectively.

### Evaluation of measles vaccination

According to the recommended schedule of expanded program on immunization in China, the first dose of MCV (MCV1) should be delivered at 8 months of age and the second dose of MCV (MCV2) between 18 and 24 months of age. In this study, we used date of birth and vaccination dates to calculate the age of immunization for each dose. Then, we evaluated the measles vaccination status of each child based on the age of immunization. Accordingly, vaccination status for MCV1 orMCV2 was classified into three categories: timely, delayed or no vaccination. Children were classified as “timely vaccination” if they received MCV1 at 8 months or MCV2 between 18 and 24 months; “delayed vaccination” if they received MCV1 later than 8 months or MCV2 later than 24 months; “no vaccination” if they had not yet received MCV1 or MCV2 at the time of interview. When considering two doses together, only children who received both doses on time were deemed as timely vaccination. Else, they would be classified as delayed vaccination or no vaccination accordingly. The coverage of MCV1 and MCV2 included timely and delayed vaccination.

### Ethics statement

This study was approved by the Institutional Review Board at Chinese Centers for Disease Control and Prevention. Signed informed consent was obtained from all the participants (children’s primary guardians) before the survey.

### Statistical analysis

Categorical variables (e.g. sociodemographic characteristics, MCV coverage and source of vaccination information) were presented as percentage (%), while delays of MCV vaccination were presented as median in days. Chi-square test was used to examine the associations of MCV1 and MCV2 vaccination. The Kaplan-Meier method was used to estimate the cumulative coverage of MCV, and Log-rank tests were used to compare the difference across subgroups. Univariate and multivariate logistic regression models were used to examine the predictors of delayed MCV1 vaccination. MapInfo 7.8 (Pitney Bowes Inc., Stamford, USA) and SPSS 20.0 (SPSS Inc, Chicago, IL, USA) were used for data analysis.

## Results

### Sociodemographic characteristics

Of the 1467 children, 63.1% were from ethnic minorities and 36.9% were Han ethnicity. The proportions of children aged 8–23 months, 24–47 months, 48–71 months and 72–84 months were 18.3%, 38.0%, 29.4% and 14.3%, respectively. Nearly 80% of primary guardians of the children were the parents and 59.3% of the guardians had middle school or higher education. 81.9% of the children received vaccination at village clinics or township health centers (Table 1).

**Table 1:** Sociodemographic characteristics of the sample (N = 1467).

		N	%
Ethnicity	Han	542	36.9
	Minorities	925	63.1
Age (months)	8-	268	18.3
	24-	557	38.0
	48-	432	29.4
	72–84	210	14.3
Primary guardians	Parents	1150	78.5
	Grandparents	317	21.5
Education levels of primary	Primary school and below	597	40.7
guardians	Middle school and above	870	59.3
Vaccination units	Village clinic	1201	81.9
	Township health center	256	17.5
	Others	10	0.6

### Coverage of MCV

The coverage of MCV1 and MCV2 were 97.5% and 93.4%, respectively, among which timely vaccination rates for MCV1 and MCV2 were 63.8% and 84.0%. Over 30% and nearly 10% of the subjects received MCV1 or MCV2 late. Taking MCV1 and MCV2 vaccination as a whole, 57.7% of the children were timely vaccinated with 35.1% delayed (Table 2).

**Table 2:** Vaccination coverage of measles containing vaccine (MCV) among the children, 2016.

	Age (months)	Total (N)	Coverage (%)	Vaccination behaviors	
				Timely (%)	Delayed (%)
MCV1	≥8	1467	97.5	63.8	33.7
MCV2	≥18	1340	93.4	84.0	9.4
MCV1+MCV2	≥18	1340	92.8	57.7	35.1

There were 495 and 126 children who received MCV1 or MCV2 late. The median delays for MCV1 and MCV2 vaccination were 46 days and 166 days, respectively (Table 3).

**Table 3:** Delay of MCV vaccination (in days).

	N	Median	Q1	Q3
MCV1	495	46	17	147
MCV2	126	166	48	396

**Fig 2:**
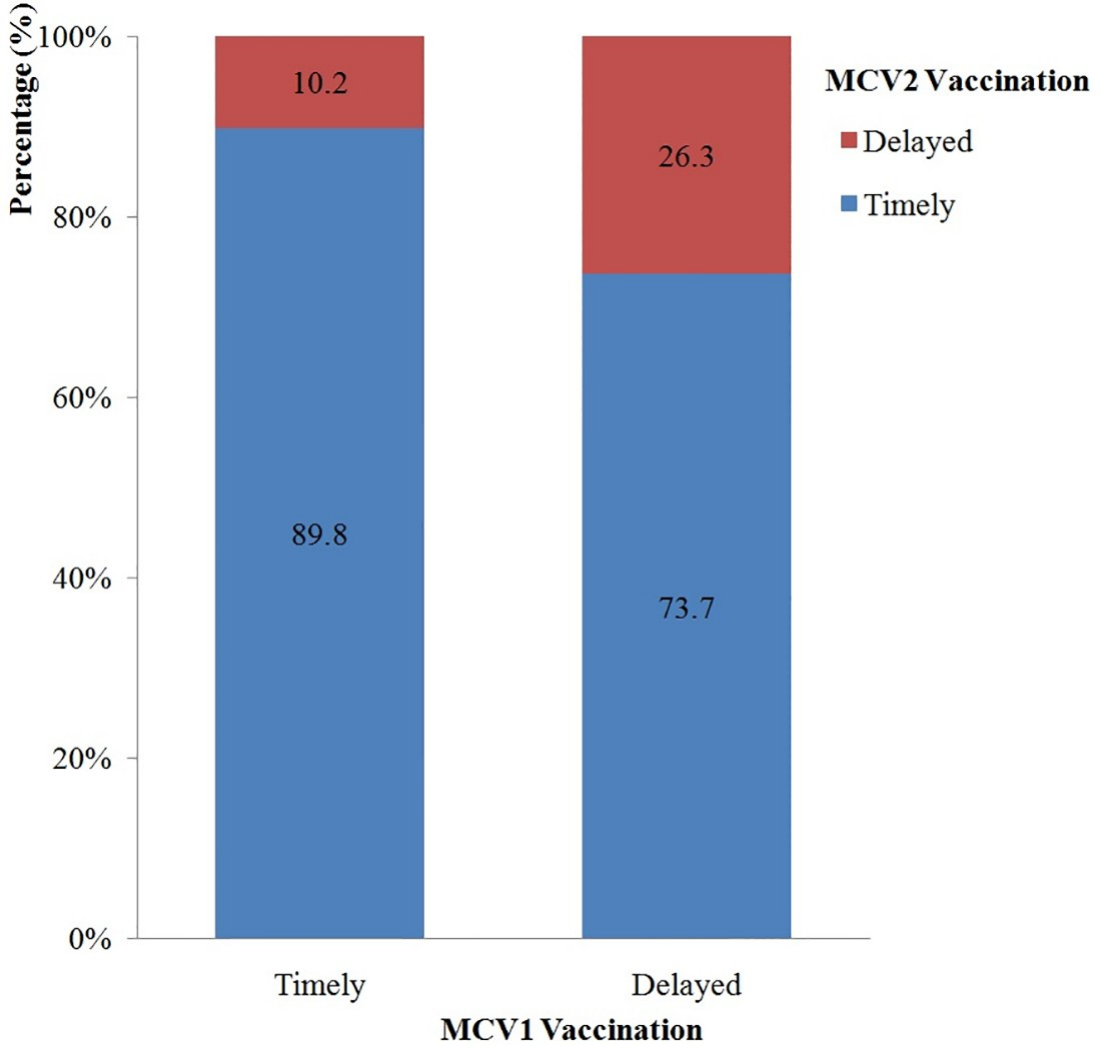
The associations between of MCV1 and MCV2 vaccination.

There were significant associations between MCV1 and MCV2 vaccinations (χ^2^ = 59.3, P<0.01). Compared to children who received MCV1 on time, children who did not received MCV1 timely were 3.1 times more likely to be delayed for MCV2 (10.2% vs. 26.3%) (Fig 2).

### Cumulative coverage of MCV

**Fig 3:**
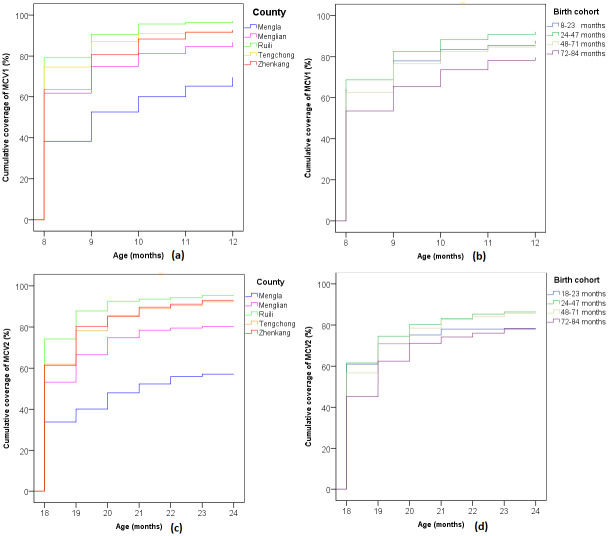
Cumulative coverage of MCV by county and birth cohort.

Fig 3 showed the cumulative coverage of MCV1 and MCV2. The observation period for MCV1 was from 8 months to 12 months of age. In Fig 3(a), there were significant differences in the cumulative coverage of MCV1 across the five border counties (Log-rank χ^2^ = 79.7, P<0.01). During the period, the cumulative coverage of MCV1 increased from 38.5% to 64.0% in Mengla, and increased from 80% to 94% in Ruili. In Fig 3(b), significant differences in the cumulative coverage were also observed across the four birth cohorts (Log-rank χ^2^ = 79.7, P<0.01). The cumulative coverage was the highest in the birth cohort of 24–47 months and the lowest in the birth cohort of 72–84 months. The observation period for MCV2 ranged from 18 months to 24 months of age. In Fig 3(c), significant differences in the cumulative coverage of MCV2 were also found across the counties (Log-rank χ^2^ = 127.4, P<0.01). Mengla and Ruili remained to be the lowest and the highest, respectively. In Fig 3(d), the cumulative coverage of MCV2 increased with age, but no significant differences were found across the four birth cohorts (Log-rank χ^2^ = 3.2, P = 0.07).

### Predictors for the delay of MCV1 vaccination

In the univariate analyses, children who were ethnic minority, and whose primary guardian had higher education level or knew the importance of immunization, were less likely to receive MCV1 late. After adjustment for county and birth cohort, the results from the multivariate logistic regression model indicated that children whose primary guardian knew the MCV schedule were less likely to receive MCV1 late (OR = 0.59, P<0.01) (Table 4).

**Table 4:** Results from the logistic regression models for the delay of MCV1 vaccination.

Variables		N	Univariate model			Final multivariate model†		
			OR	95%CI	P	OR	95%CI	P
Ethnicity	Minorities (Ref.)	925						
	Han	542	0.80	0.64–0.98	0.04	0.76	0.56–1.02	0.07
Annual income	<3835 CNY (Ref.)	745						
	≥3836 CNY	722	1.23	0.99–1.53	0.06	1.16	0.87–1.54	0.31
Guardians	Parents (Ref.)	1150						
	Grandparents	317	0.94	0.72–1.22	0.64	0.92	0.69–1.22	0.55
Education levels of guardian	Primary school and below (Ref.)	597						
	Middle school and above	870	0.78	0.62–0.96	0.02	0.95	0.74–1.21	0.67
Believing immunization is safe for children	No (Ref.)	57						
	Yes	1410	0.97	0.56–1.68	0.92	0.71	0.38–1.35	0.30
Knowing the importance of immunization	No (Ref.)	27						
	Yes	1440	0.96	0.45–2.13	0.93	0.70	0.30–1.62	0.40
Having convenient access to immunization service	No (Ref.)	93						
	Yes	1374	1.68	1.04–2.71	0.03	1.63	0.94–2.83	0.09
Knowing the schedule of MCV vaccination	No (Ref.)	485						
	Yes	982	0.57	0.45–0.72	<0.01	0.63	0.48–0.83	<0.01

OR, odds ratio; Ref, referent category.

^†^Final multivariate model after adjustment for county and birth cohort.

### Sources of information about vaccination

Results in the Table 5 show that doctors at vaccination units were the most common source of information about vaccination (86.4%), followed by TV (38.2%) and promotional materials (33.1%). Doctors at vaccination units were also the most desired source of vaccination information (Table 5).

**Table 5:** Actual and the most desired sources of vaccination information.

	Actual (%)	Desired (%)
**1. Doctors at vaccination units**	**86.4**	**72.9**
**2. TV**	**38.2**	**7.6**
**3. Promotional materials**	**33.1**	**7.6**
4. Friends and relatives	18.7	1
5. Specific trainings	13.5	2.7
6. Pediatricians	10.4	0.4
7. Mobile phone message	10.2	2.5
8. Radio	8.5	1.1
9. The Internet	6.6	1.1
10. Consulting hotlines	6.5	1
11. Newspapers	5.9	0
12. WeChat subscriptions	5.8	2.2
13. Mobile applications	1.2	0

## Discussions

This cross-sectional study assessed MCV vaccination status among children from border areas of the Southwestern China. Findings of the study can be summarized as follows. Firstly, although the coverage for MCV1 was as high as 97.5%, the proportion of timely vaccination was relatively low, especially in certain counties. Secondly, knowing the schedule of MCV vaccination among parents was the most prominent protective factor for timely vaccination. Finally, doctors at vaccination units were the main and also the most desired channel of vaccination information.

In this study, the coverage rates of MCV1 and MCV2 in children were 97.5% and 93.4%, respectively, which were relatively lower than the national average in recent years (over 99% for both MCV1 and MCV2) [11]. Similar to the reports from Italy and Brazil, the coverage of MCV2 in the Southwestern China is around 5 percentage points lower than the MCV1 coverage, and with distinct regional disparities [15, 16]. WHO recommends that in order to stop the measles transmission, the coverage rates for both the first and second doses of MCV should achieve at least 95% [17], since the second dose is critical in improving the coverage and efficacy of MCV vaccination [18]. Therefore, the coverage of the second dose of MCV vaccination should be further strengthened in border areas of Yunnan Province. Moreover, although the timeliness of MCV vaccination has not yet been included as an evaluation index of National Immunization Program in China, a growing number of studies have emphasized its importance in preventing measles transmission [19–21]. It has been reported that in the measles elimination settings, infant immunity against measles wanes earlier [22]. This implies that the delay of MCV vaccination poses a higher threat to children than before. The median delay of MCV1 vaccination in our study was close to that reported in another study from Guangxi, China, where it was 41 days [23]. However, the median delay period of MCV2 was up to 166 days. It is likely because some guardians forgot the scheduled date of MCV2 vaccination. Furthermore, we found that children who did not receive MCV1 on time were more likely to be delayed for the second dose. Thus, more attention should be gained for this group to improve the coverage of the second dose.

To understand the age pattern of MCV vaccination in border areas of Yunnan Province, the inverse Kaplan-Meier survival model was used to estimate the cumulative coverage of MCV and compared it by birth cohorts and counties. We found that the cumulative coverage in younger birth cohorts was higher than the older ones, which suggests that measles vaccination has been improved in recent years. It may be attributable to the continuous efforts to eliminate measles from China in recent years [24]. Moreover, regional disparities of vaccination appeared as a new challenge for measles elimination in many countries [15, 16]. In this study, we noticed that the age specific MCV coverage rates varied greatly across the five border counties. For example, the cumulative coverage of MCV1 in Mengla was less than 40% at 8 months and over 60% at 12 months, while the respective rates were 80% and 95% in Ruili. These results highlight the critical need to devise and implement intervention programs to eliminate the regional disparities of MCV vaccination in the border areas.

Vaccine hesitancy refers to the delay in acceptance or refusal of vaccination despite the availability of vaccination services. It is influenced by factors, such as complacency, convenience and confidence [25]. With economic and social developments, both parental awareness towards immunization and the access to immunization service in China have been improving. But in a multi-country study, the proportions of people who thought vaccines were important, safe and effective in China were still lower compared to developed countries [26]. Thus, it is of great significance to identify determinants of delayed vaccination as a perquisite to improving vaccination behaviors. Consistent with previous studies in China [27, 28], findings of this study also indicate that lower parental education level and lower household income were associated with delayed or incomplete vaccination, suggesting the key populations for measles vaccination. We also found that ethnic minority groups and people who do not have convenient access to immunization service were less likely to receive MCV vaccination on time. In the multivariate logistic regression model, knowing the MCV vaccination schedule was the only significant protective factor for the timely MCV1 vaccination. Results in other studies also revealed that parental forgetfulness was the main cause of delayed or no vaccination [29, 30]. Moreover, studies in the Western countries showed that worrying safety, effectiveness and necessity of vaccination among parents were the main factors of vaccines hesitancy, and more than half of them desire to receive additional information about the childhood vaccinations [31, 32]. For timely vaccination, it is necessary to build parental trust in health-care services and further emphasize the schedule in future vaccination education [33].

Finally, like some other studies [34, 35], our findings highlight the critical role played by rural doctors in the routine immunization services. Over 99% of the children in this study received MCV vaccination at village clinics or township health centers, and 70–80% of the guardians got or desired to get information from doctors at vaccination units. Although TV was also a main source of vaccination information in this study, several other papers showed that receiving information on vaccinations from media or the internet was associated with lower immunization rates [36], which may be due to the fact that parents may receive negative information about vaccines from Public Medias but lack enough community supports to counter it [25]. Other studies also indicated that parents were more likely to believe messages delivered by local rural doctors because they may recognize parents’ knowledge levels on vaccination and can provide more tailored information [37, 38]. Thus, more economic and technical supports should be given to rural doctors to ensure effective preventive healthcare services [39].

This study has certain limitations. First, this study was conducted only in five counties. Caution is needed when generalizing these findings to other border areas of Yunnan Province and other parts of China. Second, information about local immunization staffs, equipment and management was not collected in this study; therefore, the results cannot comprehensively reflect routine immunization services in these counties. Third, we measured vaccination status of the children exclusively by parent-held vaccination certificates or cards. Thus, the coverage of MCV vaccination may be underestimated. Despite these limitations, our study has important implications for health policymakers to improve the timeliness of MCV vaccination in border areas of Southwest China.

## Conclusions

Our study revealed that timely vaccination coverage for MCV in border areas of Yunnan Province was suboptimal and differed across counties. In addition, education on MCV immunization schedule provided by local health professionals may help to reduce delayed vaccination.

## Supporting information

S1 File (DOCX)

S1 Data (XLS)
