# Supplementary material for: Measles vaccination among children in border areas of Yunnan Province, Southwest China
Source: PLoS One. 2020 Oct 21;15(10):e0240733. doi: 10.1371/journal.pone.0240733 (PMC7577443; doi:10.1371/journal.pone.0240733)
Supplement: S1 File — (DOCX) [file pone.0240733.s001.docx]

**Questionnaire used in the study**

Dear friends, we are conducting a survey on measles vaccination among children in border areas of Yunnan Province. It is very essential for the government in improving immunization service. We assure you that all the responses are only used for academic purpose and your personal information will be kept confidentially. Thank you for your participation.

County Township Village

1. **Information of child**
   1. Date of birth (YYYYMM/DD): □□□□/□□/□□
   2. Gender : ① Male ② Female □

1.3 Ethnicity: ① Han ② Minority □

1. **Information of primary guardian**

2.1 Who is primary guardian of the child?

① Mother ② Father ③ Grandparents ④ Others □

2.2 The primary guardian’s education level

① Primary school and below ② Middle school and above □2.3 Household income per capita per year (CNY)

① <3835 ② ≥3836 □

1. **Information of MCV vaccination**

3.1 Date of MCV1 vaccination □□□□/□□/□□3.2 Date of MCV2 vaccination □□□□/□□/□□

3.3 Place of vaccination

① Village clinics/township health centers ② Other places □

1. **Guardian’s knowledge and attitudes regarding MCV vaccination**

**4.1 MCV vaccination is important for children’s health.** □

① Strongly agree ② Agree ③ Neither agree or disagree ④ Disagree ⑤ Strongly disagree

**4.2 MCV vaccination is safe for children to have.** □

① Strongly agree ② Agree ③ Neither agree or disagree ④ Disagree ⑤ Strongly disagree

**4.3 It is convenient to receive MCV vaccination.** □

① Strongly agree ② Agree ③ Neither agree or disagree ④ Disagree ⑤ Strongly disagree

**4.4 Do you know the recommended schedule of MCV vaccination** □

① YES ② NO

1. **Have you got information from the following channels?**

| 5.1  Doctors at vaccination units | YES □ | NO □ |
| --- | --- | --- |
| 2.   TV | YES □ | NO □ |
| 3.   Promotional materials | YES □ | NO □ |
| 4.   Friends and relatives | YES □ | NO □ |
| 5.   Specific trainings | YES □ | NO □ |
| 6.   Pediatricians | YES □ | NO □ |
| 7.   Mobile phone message | YES □ | NO □ |
| 8.   Radio | YES □ | NO □ |
| 9.   The Internet | YES □ | NO □ |
| 10.  Consulting hotlines | YES □ | NO □ |
| 11.  Newspapers | YES □ | NO □ |
| 12.  WeChat subscriptions | YES □ | NO □ |
| 13. Mobile applications | YES □ | NO □ |

1. **Which of the following you would prefer to get information from?**

| 5.1  Doctors at vaccination units | YES □ | NO □ |
| --- | --- | --- |
| 2.   TV | YES □ | NO □ |
| 3.   Promotional materials | YES □ | NO □ |
| 4.   Friends and relatives | YES □ | NO □ |
| 5.   Specific trainings | YES □ | NO □ |
| 6.   Pediatricians | YES □ | NO □ |
| 7.   Mobile phone message | YES □ | NO □ |
| 8.   Radio | YES □ | NO □ |
| 9.   The Internet | YES □ | NO □ |
| 10.  Consulting hotlines | YES □ | NO □ |
| 11.  Newspapers | YES □ | NO □ |
| 12.  WeChat subscriptions | YES □ | NO □ |
| 13. Mobile applications | YES □ | NO □ |

**Interviewer Signature: ___________ Signature Date: ___________**
